# Evaluation of severity in critically ill patients (cips) with limitation of life-sustaining therapy (LLST)

**DOI:** 10.1186/2197-425X-3-S1-A659

**Published:** 2015-10-01

**Authors:** J Ruiz Moreno, E González Marín, MJ Esteve Paños, S Godayol Arias, MJ Riba Ribalta, N Conesa Folch, R Corcuera Romero de la Devesa, F Baigorri González, A Artigas Raventós

**Affiliations:** QuirónSalud Hospital Universitario Sagrat Cor, Critical Care Department, Barcelona, Spain; QuirónSalud Hospital Universitario Sagrat Cor, Emergency Department, Barcelona, Spain; Hospital de Clínicas de Sabadell & QuirónSalud Hospital Universitario Sagrat Cor, Critical Care Department, Sabadell, Spain

## Introduction

It is considered that the severity of CIPs with LLSt is higher than the overall CIPs requiring ICU admission. The identification of specific clinical variables which determine the severity of the CIPs with LLST perhaps has not been researched enough.

## Objectives

- To asses the severity of the CIPs with LLST in comparison to CIPs with LLS.
- To evaluate and compare the mortality between two populations.

## Methods

- Type of study: prospective, analytical, longitudinal, and observational
- Period: January 1-2011 / June 30-2014 (42 months)
- Setting : Medical / Surgical ICU
- Population: 2559 CIPs admitted consecutively to the ICU; sample: 220 CIPs with LLST.
- Exclusión criteria: CIPs < 16 y., major burn CIPs, incomplete clinical documentation, and voluntary discharge.
- Variables analyzed:

- Age
- Hospital mortality
- Case - mix: severe sepsis, metabolic acidosis, total parenteral nutrition (TPN), oncological pathology, intra-abdominal pressure (IAP), blood products, cultures, cardiac continous output (CCO), advanced life support (ALS) before LLTS applied, FGC, FBC.

- Statistical analysis: Ji squared and contrast of means (Student's t)

## Results

See tables 1 and 2.

**Table 1:** Results 1.

	Global	% - SD	LLST	% - SD	without LLST	% - SD	p value
CIPs	2559	100	220	8,6	2339	91,4	0,0001
Age	65,9	16,7	77,2	10,6	64,8	16,8	0,0001
Mortality	182	7,1	115	52,3	67	2,96	0,0001
Sepsis	484	18,9	122	55,4	362	15,4	0,0001
Metabolic acid.	955	37,3	155	70,4	800	34,2	0,0001
oncol. pathology	903	35,3	84	38,2	819	35,0	NS
TPN	467	18,27	72	32,7	395	16,8	0,0001

**Table 2:** Results II.

	Global	% - SD	LLST	% - SD	without LLST	% - SD	p value
IAP	136	5,3	51	23,1	85	3,63	0,0001
Blood products	500	19,5	99	45,0	401	17,1	0,0001
Cultures	689	26,9	144	65,4	545	23,3	0,0001
ALS	85	3,32	16	7,3	69	2,9	0,0001
FGC	54	2,1	21	9,5	33	1,4	0,0001
FBC	61	2,4	24	10,9	37	1,6	0,0001
CCO	114	4,4	46	20,9	68	2,9	0,0001

## Conclusions

- More than 50 % of CIPs with LLST die.
- According to all clinical variables, SS, metabolic acidosis, TPN, IAP, blood products, cultures, ALS, FGC, FBC, and cardiac output are much higher in CIPs with LLST.

## References

[1] Wiegand DL, Grant MS (2014). Bioethical Issues Related to Limiting Life-sustaining Therapies in the Intensive Care Unit Disclosures. J Hospice Palliative Nursing.

[2] Wunsch H, Harrison DA, Harvey S, Rowan K (2005). End of life decisions: a cohort study of the withdrawl of all active treatment in intensive care units in the United Kingdom. Intensive Care Med.

